# Antioxidant SkQ1 delays sarcopenia-associated damage of mitochondrial ultrastructure

**DOI:** 10.18632/aging.100636

**Published:** 2014-02-05

**Authors:** Valeriya B. Vays, Chupalav M. Eldarov, Irina M. Vangely, Nataliya G. Kolosova, Lora E. Bakeeva, Vladimir P. Skulachev

**Affiliations:** ^1^ Lomonosov Moscow State University, Belozersky Institute of Physico-chemical Biology, Vorobyevy Gory, Moscow 119992, Russia; ^2^ Lomonosov Moscow State University, Institute of Mitoengineering, Vorobyevy Gory, Moscow 119992, Russia; ^3^ Institute of Cytology and Genetics, Siberian Branch of Russian Academy of Sciences, Acad., Novosibirsk 630090, Russia

**Keywords:** sarcopenia, aging, ultrastructure, mitochondria, oxidative stress, mitochondria-targeted antioxidant SkQ1

## Abstract

A comparative electron-microscopic study of ultrastructure of mitochondria in skeletal muscles of the 3- and 24-month-old Wistar and OXYS rats revealed age-dependent changes in both general organization of the mitochondrial reticulum and ultrastructure of mitochondria. The most pronounced ultrastructure changes were detected in the OXYS rats suffering from permanent oxidative stress. In the OXYS rats, significant changes in mitochondrial ultrastructure were detected already at the age of 3 months. Among them, there were the appearance of megamitochondria and reduction of cristae resulting in formation of cristae-free regions inside mitochondria. In the 24-month-old OXYS rats, mitochondrial reticulum was completely destroyed. In the isotropic region of muscle fiber, only small solitary mitochondria were present. There appeared regions of lysed myofibrils as well as vast regions filled with autophagosomes. A mitochondrial antioxidant SkQ1 (given to rats with food daily in the dose of 250 nmol/kg of body weight for 5 months beginning from the age of 19 months) prevented development of age-dependent destructive changes in both the Wistar and OXYS rats.

## INTRODUCTION

Sarcopenia, the gradual loss of muscle mass and function, is a common feature of human aging. To cure sarcopenia, it is necessary to maintain skeletal muscle homeostasis and repair. While aerobic exercises have been documented to attenuate and even partially reverse sarcopenia, a recent study reported only 2% older subjects exercised on a regular basis [1]. Therefore, there is a great need for a more available intervention to overcome sarcopenia.

The molecular mechanisms leading to sarcopenia are not completely identified, but the retardation of an oxidative damage entailed with an age-linked mitochondrial dysfunction occurring in the muscle cells looks as promising approach to treat this disease. The mitochondria are an important source of reactive oxygen species (ROS) in eukaryotic cells [2]. Mitochondrial ROS production associated with a dysfunction of respiratory chain complexes has been implicated in a number of degenerative diseases and biological aging [3-12]. As key players in apoptosis, mitochondria are assumed to be involved in the age-linked myocyte loss [13]. During the last eight years, the ability of a mitochondria-targeted antioxidant SkQ1 [10-(6'-plastoquinonyl) decyltriphenylphosphonium] to slow down aging and to retard, arrest, and in some cases even reverse the development of many age-related traits has been proved on a different animal models [2, 14-16]. However, effects of the SkQ1 on sarcopenia have not been investigated yet. Generally, there are no comprehensive investigations in mitochondrial ultrastructure during the sarcopenia development. Here we evaluated, by means of electron microscopy, the ability of the SkQ1 to retard the development of a sarcopenia-linked mitochondrial damage in Wistar and OXYS rats.

Over the last decade, a large amount of experimental data has been obtained which demonstrated that senescence-accelerated OXYS rats are a good model for studies of mechanisms of age-related stress-linked oxidative alterations as well as an assay of new therapeutic approaches. OXYS rats show a very early development of age-associated pathological phenotypes similar to several geriatric disorders observed in humans, including cataract, AMD-like retinopathy, osteoporosis and age-related cerebral dysfunctions [16-21]. It was hypothesized that the accelerated senescence of OXYS rats associates with a ROS-induced progressive mitochondrial dysfunction. Therefore, dietary supplementation with antioxidants might prevent the premature deterioration of mitochondrial function in OXYS rats [22]. Recently, we showed that very low doses of SkQ1 prevent some consequences of accelerated senescence in these rats. In particular, SkQ1 completely prevented or at least retarded the development of cataract and retinopathy [15, 23], an age-dependent decline of the immune system [15, 24], the sexually motivated behavior [25] and some other behavioral alterations [16, 19] occurring in the middle-age OXYS rats.

The aim of this study was to examine ultrastructural characteristics of mitochondrial apparatus of the skeletal muscle fibers of Wistar and OXYS rats during aging and to investigate possible effects of SkQ1 on the sarcopenia-linked changes of mitochondria in these rats.

## RESULTS

**Figure 1:**
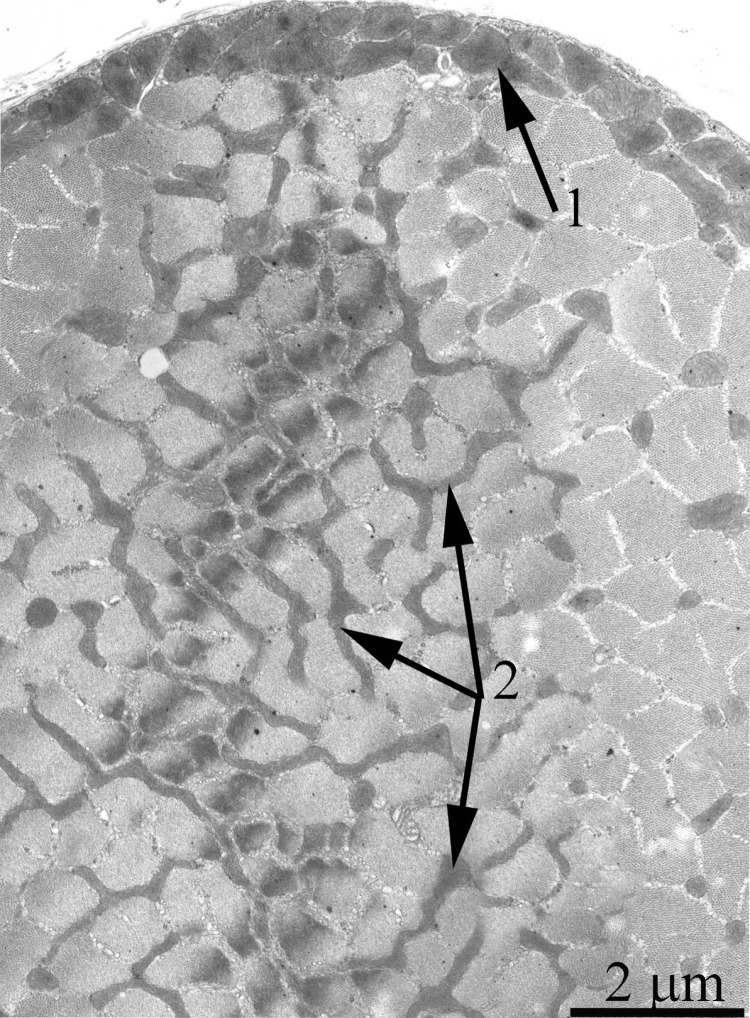
Cross-section of a muscle fiber of a 3-month-old Wistar rat. The arrows indicate: subsarcolemmal population of mitochondria (1); interfibrillar stretched mitochondria (2).

**Figure 2:**
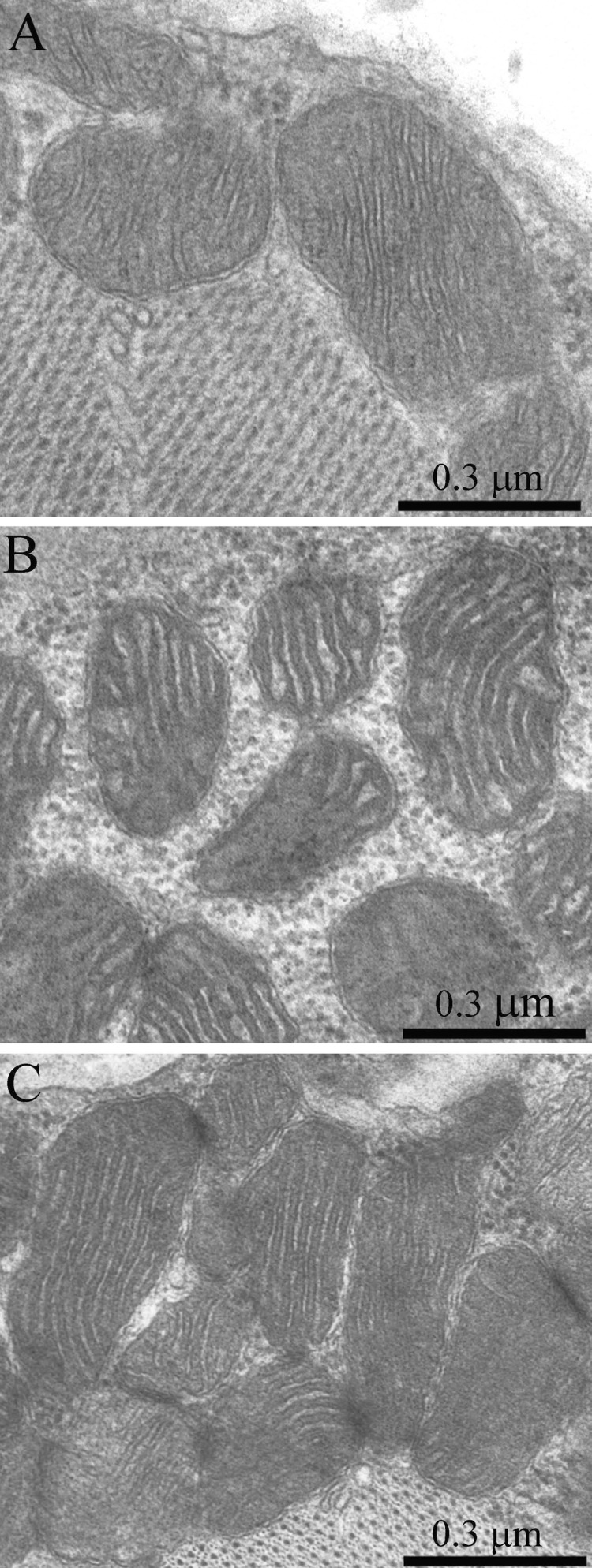
The ultrastructure of skeletal muscle mitochondria of Wistar rats of the age of 3 months (**A**), of 24 months (**B**); of 24 months treated with SkQ1 (250 nmol/kg) for the last 5 months (**C**).

As was previously shown using three-dimensional reconstitution of ultrathin serial sections [26-29], the mitochondrial profiles in skeletal muscles are united to the mitochondrial reticulum stretching along the muscle fiber. Fig. 1 presents a cross-section of a skeletal muscle fiber of the young Wistar rat. One can see a well-developed network of the mitochondrial reticulum consisting of a subsarcolemmal population of mitochondria (the arrow 1) and an interfibrillar system of stretched mitochondria (the arrows 2). The inner ultrastructure of mitochondria (Fig. 2A) revealed the cristae arranged in mutually parallel manner.

**Figure 3:**
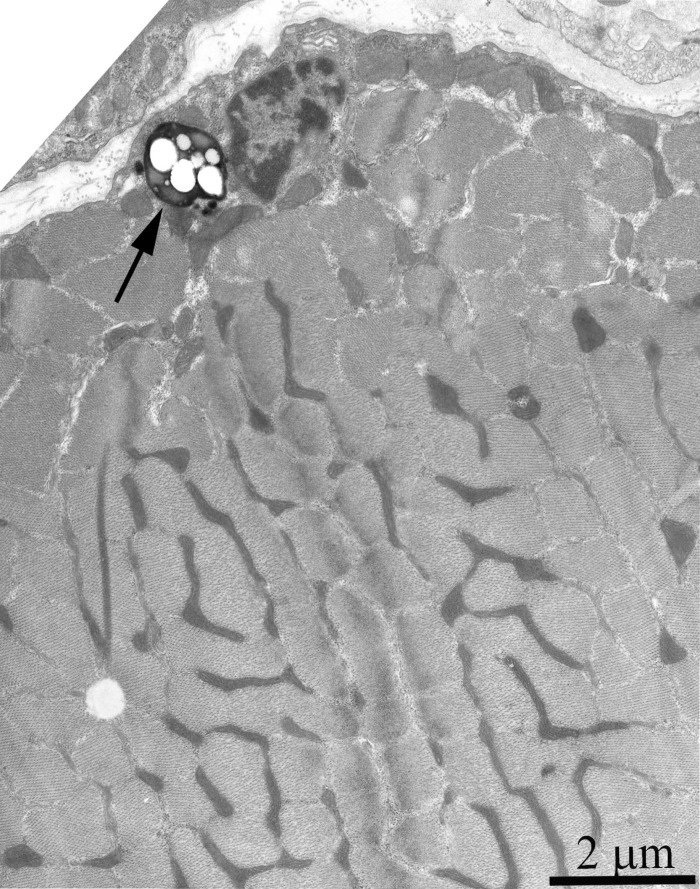
Cross-section of the muscle fiber from a 24-month-old Wistar rat. The arrow indicate autophagosome.

Ultrastructure of the mitochondrial apparatus of skeletal muscle of the Wistar rats at the age of 24 months proved to be different from that of young animals. First of all, in clusters of subsarcolemmal mitochondria, one could see autophagosomes surrounded by mitochondria (see, e.g., Fig. 3, the arrow). As to the isotropic region, only slightly branched mitochondria were revealed instead of the network of elongated mitochondria. Mitochondrial ultrastructure was also significantly changed (Fig. 2B): the electron density of the matrix is increased alongside with a decrease in its volume, the intermembrane (intracristal) space being increased. According to the David Green's classification [30], such changes in the mitochondria ultrastructure correspond to the de-energized state of mitochondria.

**Figure 4:**
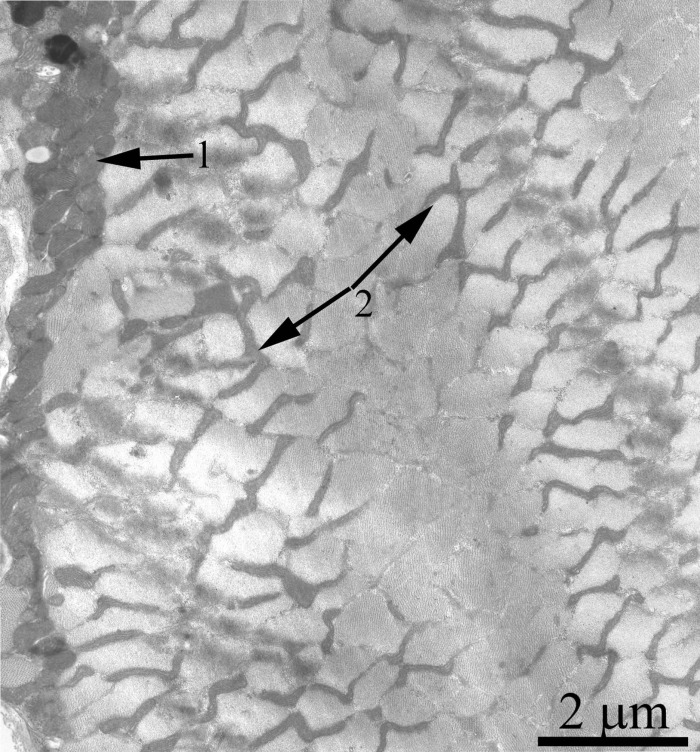
A fragment of the muscle fiber from a 24-month-old Wistar rat treated with SkQ1. The arrows indicate: subsarcolemmal population of mitochondria (1) and interfibrillar stretched mitochondria (2).

Fig. 4 shows a general view of a skeletal muscle fiber of the SkQ1-treated Wistar rats at the age of 24 months. The mitochondrial apparatus of these animals looks well developed. One can see a large subsarcolemmal mitochondrial cluster (Fig. 4, the arrow 1) and a numerous branched interfibrillar mitochondrial profiles (Fig. 4, the arrow 2). The mitochondrial ultrastructure proved to be intermediate between those of young and old SkQ1-untreated animals (Fig. 2C).

The results were processed mathematically using stereotypic methods. For each of the three groups of Wistar rats (3-month-old, 24-month-old, 24-month-old SkQ1-treated rats), we calculated the mitochondria-occupied portion of isotropic region. Statistic analysis embraced fifty photos for each group of the animals. Fig. 12D (see below) showed results of such an analysis. It was found that this portion in the Wistar rats decreased from 38±2 % in young animals to 24±1,5 % in old animals. In the old Wistar rats treated with SkQ1, this value proved to be equal to 33±4 %, i.e. closer to that of the young than of the old animals.

**Figure 5:**
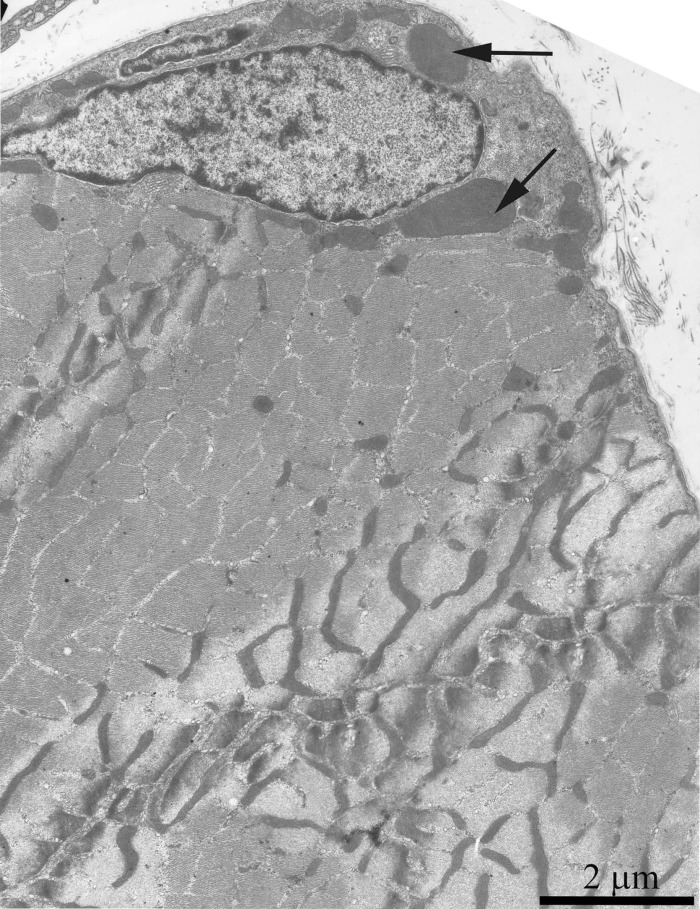
Cross-section of the muscle fiber of a three-month-old OXYS rat. The arrows indicate mitochondria which are significantly larger than the usual organelles.

**Figure 6:**
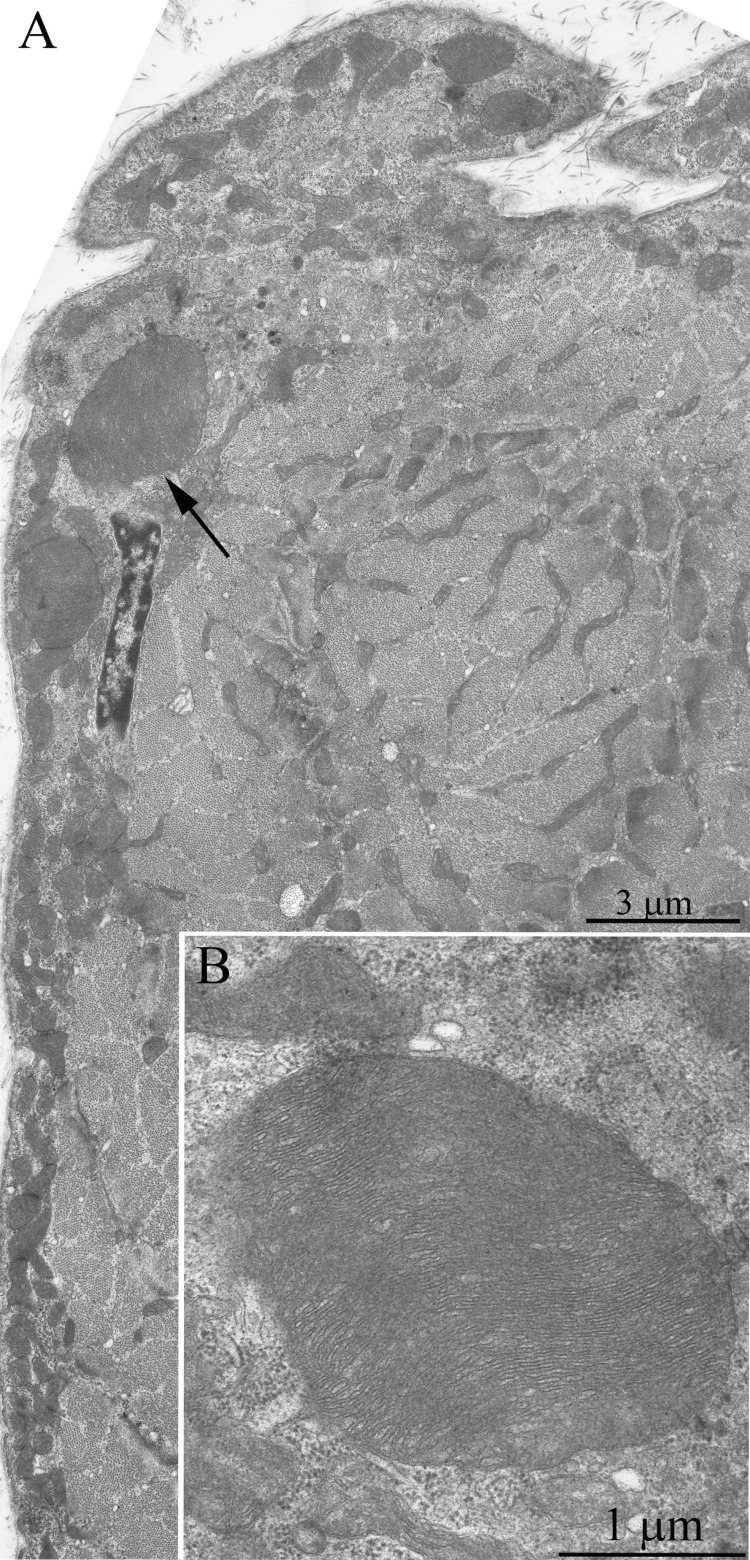
(**A**) Cross-section of the muscle fiber of a three-month-old OXYS rat. See large mitochondria in the nucleous region (one of them is indicated with the arrow). (**B**) a large mitochondrion at higher magnification.

**Figure 7:**
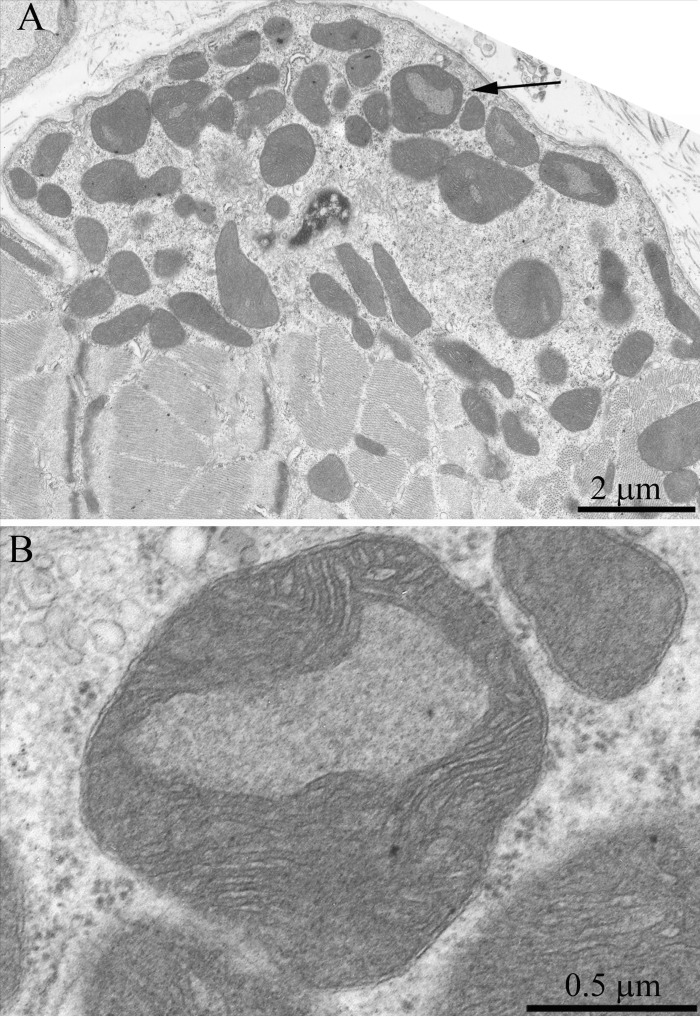
(**A**) Subsarcolemmal population of mitochondria of a three-month-old OXYS rat. The arrow indicates the mitochondrion presented in (**B**) at higher magnification. One can see a large cristae-free region occupied by a homogenous content of a low electron density.

**Figure 8:**
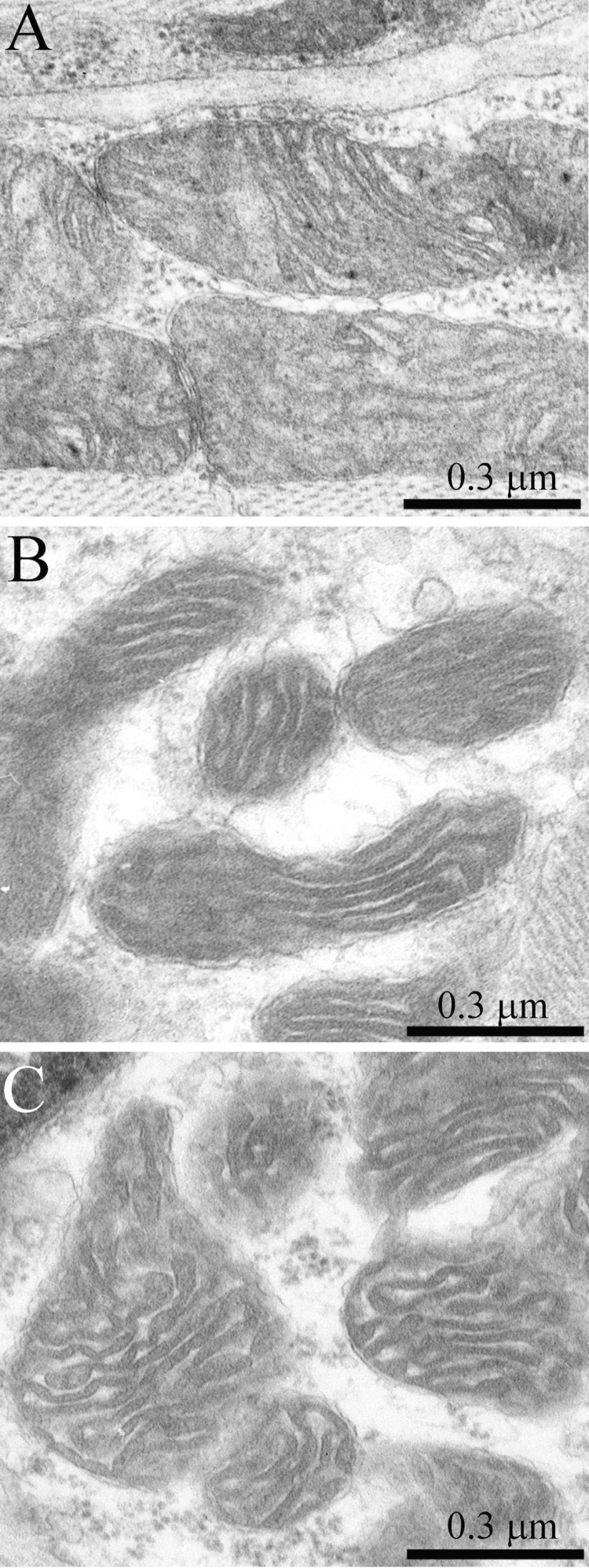
The ultrastructure of skeletal muscle mitochondria of OXYS rats of the age of three months (**A**), 24 months (**B**); 24 months treated with SkQ1(250 nmol/kg) for the last 5 months (**C**).

The structure of muscle fiber chondriome from OXYS line rats was essentially different from that of the Wistar rats of the same age. Figs.5 and 6 present a cross-section of the muscle fiber from a 3-month-old OXYS rat. In the isotropic region, there is a network of stretched convoluted mitochondria. However, diameter of mitochondria located near the nucleous was significantly larger than in the Wistar rats (Figs. 5, 6, arrows). In the literature such mitochondria are considered as a special type of these organelles, i.e. megamitochondria, which appear in the case of various diseases or stress conditions. Changes were also observed in the ultrasctucture of subsarcolemmal mitochondria. Fig. 7 shows large intramitochondrial cristae-free areas of a low electron density in the young OXYS rats. In mitochondria retaining more usual ultrastructure, the number of cristae was reduced, the intermembrane space was enlarged, and the electron density of the matrix was decreased (Fig. 8A).

**Figure 9:**
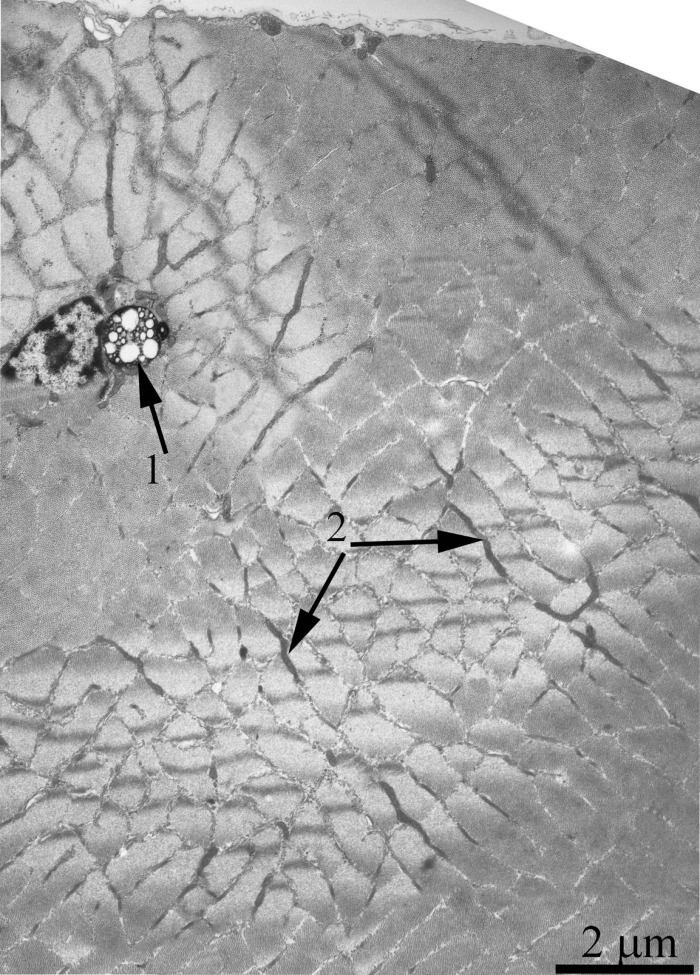
Cross-section of the muscle fiber from a 24-month-old OXYS rat. The arrows indicate: autophagosome (1) and small elongated mitochondrial profiles in the isotropic region (2).

**Figure 10:**
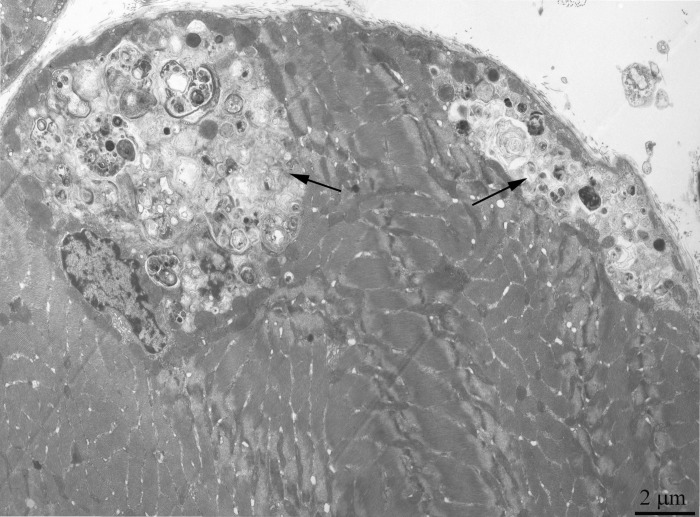
Cross-section of the muscle fiber from a 24-month-old OXYS rat. The arrows, regions occupied with autophagosomes.

Alterations in the ultrastructure of the skeletal muscle chondriome of the OXYS rats become ever strongly pronounced by the age of 24 months. Fig. 9 clearly demonstrates a dramatic degradation of the system of branched interfibrillar mitochondria. In the isotropic region, there are only small solitary mitochondria. In muscle fibers of this group of animals, we have also found another type of destructive changes in the ultrastructure of subsarcolemmal mitochondria and the whole muscle fibers. In Fig. 10, one can see extensive area occupied with autophagosomes (the arrows).

In addition to the above-described alterations in the general structure of the mitochondrial apparatus of muscle fibers, the 24-month-old OXYS rats showed significant changes in the ultrastructure of mitochondria. It consisted in a compressed electron-dense matrix with separate regions of tightly located membranes of the adjoining cristae as well as in an increased volume of the intermembrane space (Fig. 8B) that corresponds to the de-energized state.

**Figure 11:**
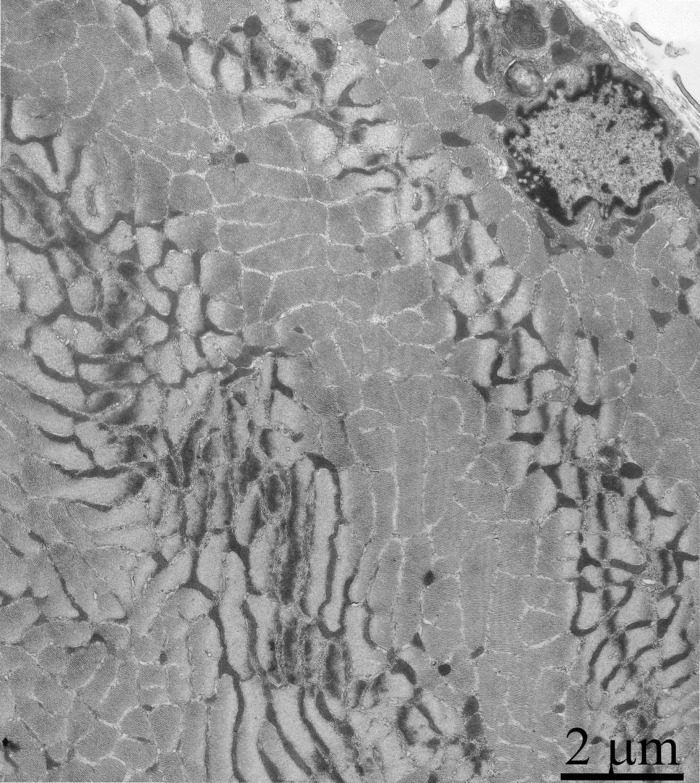
Cross-section of the muscle fiber of a three-month-old OXYS rat treated with SkQ1 (250 nmol/kg) for the last 5 months.

In the 24-month-old SkQ1-treated OXYS rats no pathological changes in the mitochondrial profiles were found (Fig. 11). In the muscle fiber, both a well-developed network of stretched branched interfibrillary mitochondria and clusters of subsarcolemmal mitochondria were seen. The inner ultrastructure of the muscle fiber mitochondria of this group of animals could be defined as the energized and “twisted” (Fig. 8C). Cristae lose their plane-parallel organization and form tubular convoluted structuresdescribed by Green et al., (1968)[30].

**Figure 12:**
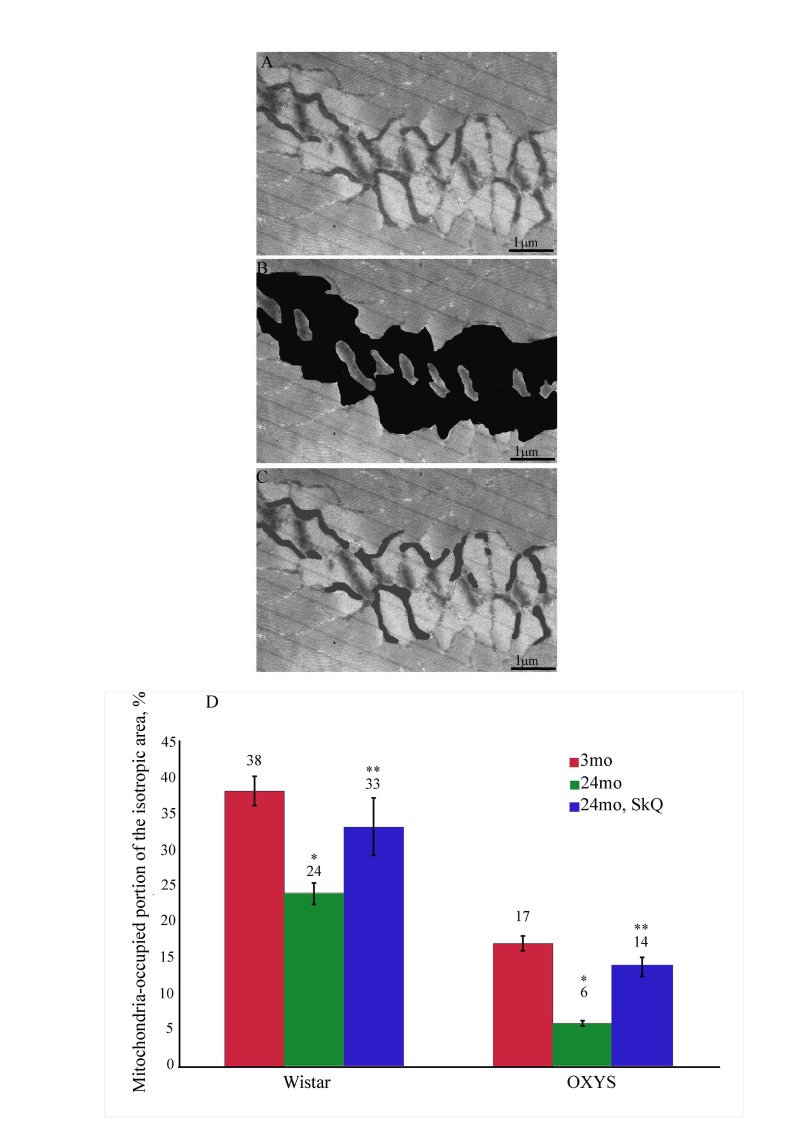
Statistic analysis of the volume occupied by the skeletal muscle mitochondria: (**A**) ultrastructure of the muscle fiber near the isotropic region; (**B**) the isotropic region near the Z-disc is stained black; (**C**) mitochondria located in the isotropic region are stained gray; (**D**) The area occupied by mitochondria located within the isotropic region as % of the total isotropic area in the Wistar and OXYS rats; (*) and (**), p<0.05 for effects of age and SkQ1, respectively.

Mathematical processing and statistical analysis of electron microphotographs of muscle fibers of OXYS rats showed (Fig. 12D) even in young animals mitochondria occupied as small as 17±1 % isotropic region. This value decreased further down to 6±0.5 % in the old OXYS rats. This effect of aging was decreased by SkQ1 (11±1 %).

## DISCUSSION

Our study of skeletal muscles of the Wistar rats have revealed age-related changes in the amount of mitochondria, forms of mitochondrial profiles and ultrastructure. In the progeric OXYS rats, destructions of the muscle tissue mitochondrial apparatus appear already by the age of 3 months. The young OXYS animals seem to be characterized by absence of a united system of the mitochondrial reticulum inherent in the young Wistar rats, mitochondrial ultrastructure indicating a decrease in coupling of respiration and ATP synthesis and small amount of interfibrilar mitochondria which occupy a smaller part of the isotropic area than even in the old Wistar rats. The situation in the OXYS rat changes from bad to ever worse during aging.

The treatment of animals with a mitochondria-targeted antioxidant SkQ1 retarded development of age-related destructive pathological changes in mitochondria of both Wistar and OXYS rats. Again, this is true for the amount of mitochondria, the development of mitochondrial reticulum and ultrastructure of the mitochondrial cristae.

It should be noted that the treatment with SkQ1 prevented development of irreversible pathological changes in the ultrastructure of whole muscle fibers, such as disassembly of myofibrils and an increase in the amount of autophagosomes. Areas of serious pathological changes are more seldom in the skeletal muscle tissue of the SkQ1-treated OXYS rats compared to the non-treated animals.

Thus, the organization of the mitochondrial profile in the skeletal muscle of Wistar rats at the age of three months is in line with the assumption that in this tissue mitochondria are united to mitochondrial reticulum. However, in the 24 months-old Wistar rats, the mitochondrial reticulum system looks degraded, and the ultrastructure of mitochondrial cristae is shifted to a de-energized state which is inherent in mitochondria incapable of transforming the energy of oxidative reactions to ATP. In the SkQ1-treated 24-month-old Wistar rats, large subsarcolemmal mitochondrial clusters and a well-developed system of interfibrillar mitochondria are observed, which resemble that in the young Wistar rats. In OXYS rats, disorders in the muscle tissue mitochondrial apparatus appear already at the age of 3 months and by the age of 24 months hypoplasia and atrophy of skeletal muscles are developed. These pathological changes are found to be prevented to a large degree by SkQ1.

Accumulating evidence supports the existence of a close relationship between declining anabolic hormones, such as growth hormone (GH) and insulin-like growth factor-1 (IGF-1) levels and age-related changes in body composition and function. Therefore, the age-dependent decline of GH and IGF-1 serum levels might promote the loss of muscle mass and strength. We recently measured the levels of these hormones in the SkQ1-treated animals [31]. It was found that an SkQ1 treatment between the ages of 19 and 23 months increased the blood levels of GH and IGF-I in the Wistar and the OXYS rats above those found in the 19 month-old animals. These results suggest that the effect of the SkQ1 against sarcopenia may be partially mediated by an activation of somatotropic (GH/IGF-1) signaling which is reduced in OXYS rats since a young age [31].

Our data characterize the OXYS rats as a good model for sarcopenia. They indicate that the treatment with low doses of mitochondria-targeted antioxidant SkQ1, even in already aged organisms, may be a promising strategy to maintain health and retard the aging process ^1^1When this paper was already prepared to be submitted, an article by D.A. Sinclair and coworkers appeared in the Cell (*A.P. Gomes et al., Declining NAD(+) Induces a Pseudohypoxic State Disrupting Nuclear-Mitochondrial Communication during Aging, Cell 155, 1624-1638, 2013*). The authors reported that aging of skeletal muscles is accompanied by strong decline in the amount of mitochondrial DNA-encoded subunits of respiratory phosphorylation – catalyzing complexes, as well as a decrease in the ATP level, amount of mitochondrial DNA and a mitochondria-occupied volume in this tissue. Such effects were mediated by a decrease in the intracellular concentration of NAD^+^, causing a decrease in SIRT1 and strong elevation of hypoxia-inducible factor 1α (Hif-1α). The effects were *in vivo* prevented by caloric restriction or injection of large amount of a NAD^+^ precursor, nicotinamide mononucleotide. Excision of SIRT1 in young animals mimicked effect of aging, including elevation of mitochondrial reactive oxygen species. All these observations are in line with our data on dramatic degradation of mitochondrial reticulum during development of sarcopenia in the skeletal muscles during aging and its prevention and reversal by mitochondria-targeted antioxidant SkQ1..

## MATERIALS AND METHODS

### Animals and diet

All procedures on the animals were carried out in accordance with European Communities Council Directive No. 86/609/EES. Male senescence-accelerated OXYS and age-matched male Wistar rats were obtained from the Shared Center for Genetic Resources of the Institute of Cytology and Genetics (ICG), Siberian Branch of the Russian Academy of Sciences (Novosibirsk, Russia). The OXYS rat strain was established based on Wistar rat strain at the Institute of Cytology and Genetics as described earlier [22] and registered in the Rat Genome Database (http://rgd.mcw.edu/). At the age of 4 weeks, the pups were taken away from their mothers, housed in groups of five animals per cage (57×36×20 cm) and kept under standard laboratory conditions (at 22± 2°C, 60% relative humidity, and natural light), provided with a standard rodent feed, PK-120-1, Ltd. (Laboratorsnab, Russia). The SkQ1 treatment (250 nmol/kg body weight daily with food) was initiated at the age of 19 months. At this age, the muscle power of the OXYS rats normalized for body weight was already reduced by 22%, compared with the Wistar (p<0.001). Starting from the age of 19 months OXYS and Wistar rats were randomly divided into two groups, i.e. control diet and diet supplemented with SkQ1 (synthesized as described earlier [14]). At the age of 24 months, the electron microscopic study was carried out, using rats at the age of 3 months as a control.

The body weight was measured before the start of treatment and at the end of experiment. OXYS rats had lower body weight in comparison with Wistar rats (respectively, 612±18 and 404±9 g at the age of 19 months). At the age of 24 months, the weights of the Wistar rats were 560±27 g without SkQ1 and 642±20 g with SkQ1. As to the OXYS rats, these values were 393±8 g without SkQ1 and 390±8 g with SkQ1.

### Electron microscopy

For electron microscopic investigation, *M. gracilis* and medial ventrum of *M. quadriceps femoris* were taken from the medial surface of the right hind leg. The sample was fixed with a 3% glutaraldehyde solution (pH 7.4) for 2 h at 4°C, then overfixed with 1% osmium tetraoxide solution for 1.5 h, and dehydrated in alcohol series with increasing alcohol concentrations (70% alcohol was saturated with uranyl acetate). The sample was embedded in an Epon-812 epoxy resin.

Serial ultrathin sections were made with a Leica ULTRACUT UCT microtome and stained by lead according to Reinolds. The resulting preparations were scanned and photographed using an H-12 electron microscope (Hitachi, Japan).

### Mathematical processing and statistical analysis

Branched mitochondrial reticulum located in the muscle fiber isotropic region has been studied. To do this, electron-transparent regions (isotropic areas of the muscle fiber) were found on electron microphotos of skeletal muscle (50 photos for each group of the animals). Then all mitochondria located in these regions were painted (see below, Fig. 12). The photos were processed with an Adobe® Photoshop® and for each photo the following two parameters were determined, i.e. the total area of the muscle fiber isotropic area (electron-transparent regions on the photos) and the total area of mitochondria located in the electron-transparent isotropic areas. Then a mitochondria-occupied portion of isotropic region was calculated. All statistical data were calculated using STATISTICA® package.

## References

[R1] Burks TN, Cohn RD (2011). One size may not fit all: anti-aging therapies and sarcopenia. Aging (Albany NY).

[R2] Skulachev VP (2013). Cationic antioxidants as a powerful tool against mitochondrial oxidative stress. Biochem Biophys Res Commun.

[R3] Fiskum G, Rosenthal RE, Vereczki V, Martin E, Hoffman GE, Chinopoulos C, Kowaltowski A (2004). Protection against ischemic brain injury by inhibition of mitochondrial oxidative stress. J Bioenerg Biomembr.

[R4] Murphy AN, Fiskum G, Beal MF (1999). Mitochondria in neurodegeneration: bioenergetic function in cell life and death. J Cereb Blood Flow Metab.

[R5] Lenaz G (2001). The mitochondrial production of reactive oxygen species: mechanisms and implications in human pathology. IUBMB Life.

[R6] Dawson TM, Dawson VL (2003). Molecular pathways of neurodegeneration in Parkinson's disease. Science.

[R7] Skulachev VP, Nystrom T, Osiewacz H.D (2003). Aging and programmed death phenomena.

[R8] Beal MF (2004). Mitochondrial dysfunction and oxidative damage in Alzheimer's and Parkinson's diseases and coenzyme Q10 as a potential treatment. J Bioenerg Biomembr.

[R9] Bossy-Wetzel E, Schwarzenbacher R, Lipton SA (2004). Molecular pathways to neurodegeneration. Nat Med.

[R10] Shen J, Cookson MR (2004). Mitochondria and dopamine: new insights into recessive parkinsonism. Neuron.

[R11] Longo VD, Mitteldorf J, Skulachev VP (2005). Programmed and altruistic ageing. Nat Rev Genet.

[R12] Skulachev VP (2012). What is “phenoptosis” and how to fight it?. Biochemistry (Mosc).

[R13] Marzetti E, Hwang JC, Lees HA, Wohlgemuth SE, Dupont-Versteegden EE, Carter CS, Bernabei R, Leeuwenburgh C (2010). Mitochondrial death effectors: relevance to sarcopenia and disuse muscle atrophy. Biochim Biophys Acta.

[R14] Antonenko YN, Avetisyan AV, Bakeeva LE, Chernyak BV, Chertkov VA, Domnina LV, Ivanova OY, Izyumov DS, Khailova LS, Klishin SS, Korshunova GA, Lyamzaev KG, Muntyan MS (2008). Mitochondria-targeted plastoquinone derivatives as tools to interrupt execution of the aging program. 1. Cationic plastoquinone derivatives: synthesis and in vitro studies. Biochemistry (Mosc).

[R15] Skulachev VP, Anisimov VN, Antonenko YN, Bakeeva LE, Chernyak BV, Erichev VP, Filenko OF, Kalinina NI, Kapelko VI, Kolosova NG, Kopnin BP, Korshunova GA, Lichinitser MR (2009). An attempt to prevent senescence: a mitochondrial approach. Biochim Biophys Acta.

[R16] Skulachev VP (2012). Mitochondria-targeted antioxidants as promising drugs for treatment of age-related brain diseases. J Alzheimers Dis.

[R17] Rumyantseva YV, Fursova A, Fedoseeva LA, Kolosova NG (2008). Changes in physicochemical parameters and alpha-crystallin expression in the lens during cataract development in OXYS rats. 5. Biochemistry (Mosc).

[R18] Zhdankina AA, Fursova A, Logvinov SV, Kolosova NG (2008). Clinical and morphological characteristics of chorioretinal degeneration in early aging OXYS rats. Bull Exp Biol Med.

[R19] Stefanova NA, Fursova A, Kolosova NG (2010). Behavioral effects induced by mitochondria-targeted antioxidant SkQ1 in Wistar and senescence-accelerated OXYS rats. J Alzheimers Dis.

[R20] Markovets AM, Saprunova VB, Zhdankina AA, Fursova A, Bakeeva LE, Kolosova NG (2011). Alterations of retinal pigment epithelium cause AMD-like retinopathy in senescence-accelerated OXYS rats. Aging (Albany NY).

[R21] Kozhevnikova OS, Korbolina EE, Ershov NI, Kolosova NG (2013). Rat retinal transcriptome: effects of aging and AMD-like retinopathy. Cell Cycle.

[R22] Shabalina IG, Kolosova NG, Grishanova A, Solov'ev VN, Salganik RI, Solov'eva NA (1995). Oxidative phosphorylation activity, F0F1-ATPase and level of liver mitochondrial cytochromes in rats with congenitally increased ability for free radical formation. Biochemistry (Mosc).

[R23] Neroev VV, Archipova MM, Bakeeva LE, Fursova A, Grigorian EN, Grishanova AY, Iomdina EN, Ivashchenko Zh N, Katargina LA, Khoroshilova-Maslova IP, Kilina OV, Kolosova NG, Kopenkin EP (2008). Mitochondria-targeted plastoquinone derivatives as tools to interrupt execution of the aging program. 4. Age-related eye disease. SkQ1 returns vision to blind animals. Biochemistry (Mosc).

[R24] Obukhova LA, Skulachev V.P, Kolosova N.G (2009). Mitochondria-targeted antioxidant SkQ1 inhibits age-dependent involution of the thymus in normal and senescence-prone rats. Aging (Albany NY).

[R25] Amstislavskaya TG, Maslova LN, Gladkikh DV, Belousova II, Stefanova NA, Kolosova NG (2010). Effects of the mitochondria-targeted antioxidant SkQ1 on sexually motivated behavior in male rats. Pharmacol Biochem Behav.

[R26] Gauthier GF, Padykula HA (1966). Cytological studies of fiber types in skeletal muscle. A comparative study of the mammalian diaphragm. J Cell Biol.

[R27] Bubenzer HJ (1966). Die Dunnen und die Dicken Muskelfasern des Swerchfels der Ratte. Z Zellforsch Mikrosk Anat.

[R28] Bubenzer HJ (1967). Die Strukturen der Skelettmuskelfasern im Hindblock auf ihre Funktionen. Umschau.

[R29] Bakeeva LE, Chentsov Yu S, Skulachev VP (1978). Mitochondrial framework (reticulum mitochondriale) in rat diaphragm muscle. Biochim Biophys Acta.

[R30] Green DE, Asai J, Harris RA, Penniston JT (1968). Conformational basis of energy transformations in membrane systems. 3. Configurational changes in the mitochondrial inner membrane induced by changes in functional states. Arch Biochem Biophys.

[R31] Kolosova NG, Stefanova NA, Muraleva NA, Skulachev VP (2012). The mitochondria-targeted antioxidant SkQ1 but not N-acetylcysteine reverses aging-related biomarkers in rats. Aging (Albany NY).

